# The role and clinical significance of DNA damage response and repair pathways in primary brain tumors

**DOI:** 10.1186/2045-3701-3-10

**Published:** 2013-02-06

**Authors:** Wil L Santivasi, Fen Xia

**Affiliations:** 1 Department of Radiation Oncology, The Ohio State University College of Medicine, 072A Starling Loving Hall, 300 W. 10th Avenue, Columbus, OH 43210, USA

**Keywords:** Brain tumor, DNA repair, DNA damage, Homologous recombination (HR), Non-homologous end-joining (NHEJ)

## Abstract

Primary brain tumors, in particular, glioblastoma multiforme (GBM), continue to have dismal survivability despite advances in treating other neoplasms. The goal of new anti-glioma therapy development is to increase their therapeutic ratios by enhancing tumor control and/or decreasing the severity and incidence of side effects. Because radiotherapy and most chemotherapy agents rely on DNA damage, the cell’s DNA damage repair and response (DRR) pathways may hold the key to new therapeutic strategies. DNA double-strand breaks (DSBs) generated by ionizing radiation and chemotherapeutic agents are the most lethal form of damage, and are repaired via either homologous recombination (HR) or non-homologous end-joining (NHEJ) pathways. Understanding and exploitation of the differences in the use of these repair pathways between tumor and normal brain cells will allow for an increase in tumor cell killing and decreased normal tissue damage. A literature review and discussion on new strategies which can improve the anti-glioma therapeutic ratio by differentially targeting HR and NHEJ function in tumor and normal neuronal tissues is the focus of this article.

## Introduction

### Primary brain tumors

In 2012, it is predicted that nearly 23,000 new primary brain tumors will be diagnosed, [1] of which 70% will be gliomas [2]. The most common type of glioma is glioblastoma multiforme (GBM), which accounts for 54% of all gliomas (42% of all primary brain tumors). Five-year survival is a dismal 4.70% for patients with GBM, [3] with a median survival time of just over 3 months following resection [4]. Despite a massive research effort, outcomes remain dismal in malignant brain tumors.

Our limited understanding of the mechanisms which underlie brain tumorigenesis severely limit preventative and therapeutic options for patients. The current standard treatment for GBM involves surgical resection followed by adjuvant radiotherapy (RT), with or without concomitant chemotherapy [5]. Disappointingly, this regimen only affords GBM patients a median survival benefit of 14.6 months- a 12 month improvement over resection alone [4,6]. It is important to note that radio- and chemotherapies are, at the molecular level, based on inducing enough DNA damage in the tumor cell to result in lethality. Unfortunately, these therapies also cause DNA damage to surrounding neuronal tissue, resulting in a variety of local and systemic toxicities. With regard to ionizing radiation (IR) treatments to the brain, side effects can be severe and include nausea, vomiting, seizure, and permanent cognitive and focal neurological deficits [7].

As such, there is a substantial research effort seeking to discover new therapeutic regimens that maximize tumor killing while minimizing these normal tissue toxicities, based on understanding of the differences in the behaviors and pathways of healthy and neoplastic cells. Current research and understanding of DNA damage response and repair (DRR) in glioma tumorigenesis and treatment response is the focus of this review.

### DNA double-strand breaks (DSBs)

A critical feature of the eukaryotic cell is its ability to maintain genome stability across generations, attributed, in part, to the sophisticated and precisely regulated DNA lesion-specific repair mechanisms. Deficiencies in DRR have been widely associated with a number of astrocytoma subtypes [8]. The pathways by which cells rectify DSBs are of particular note, as one unrepaired DSB can trigger apoptosis [9]. Erroneous repair of DSBs leads to gross genomic rearrangement, which can result in genomic instability and tumorigenesis. One example is the KIAA1549-RAF gene fusion generated by misrepair of DSBs found in pediatric astrocytomas [10]. As such, efficient and faithful DSB repair is critical to normal cell function and the prevention of neoplastic transformation in brain.

It has been well established that, in mammalian cells, DSBs are repaired through at least two distinct pathways: homologous recombination (HR) and non-homologous end-joining (NHEJ). Both repair mechanisms have implications in tumorigenesis, impact tumor response to current treatment, as well as potentially serve as therapeutic targets in brain tumor management.

## Homologous recombination (HR)

HR is a critical pathway for accurate repair of DSBs and maintenance of genomic stability. HR-mediated repair is characterized by deriving of the correct sequence from a homologous strand of intact DNA. This modeling process allows for high-fidelity repair of DSBs- much more so than repairs via NHEJ [11]. It is the primary pathway of DSB repair during the S and G2 phases of the cell cycle, in part due to the availability of sister chromatids to be used as repair templates [12].

HR mediated DSB repair follows a general scheme of nuclease-mediated resection of damaged DNA ends, polymerization of new DNA, and ligation to restore strand integrity. Detailed biochemistry of the pathway has been well described by others [13]. One of the important and well-studied proteins in the HR pathway is BRCA1, a tumor suppressor. BRCA1 serves as a “master controller” of HR, binding and regulating many downstream affectors including Mre11-Rad50-NBS1 (MRN) complex [14]. The MRN complex binds to DNA ends, and recruits nucleases, including Eme1, to clean up damaged bases and initiate the HR process [15,16]. The binding of MRN also activates ATM, which in turn phosphorylates and activates BRCA1 function in activating cell cycle checkpoints. This prevents the cell from entering mitosis before damage is repaired [17]. BRCA1 also indirectly interacts with the key HR effector protein Rad51. Rad51 and its paralogs such as XRCC3 are critical in homology searching to identify a homologous sequence on the sister chromatid [18,19]. When a homologous sequence is found, there is an exchange of the damaged strands, such that each strand is now paired with a homologous template. The damaged strands are then extended and ligated, restoring the original double-strand [20,21].

HR is a critical pathway for DSB repair fidelity. As such, dysregulation of HR processes resulting from functional deficiency of HR proteins has been associated with glioma development in many instances [8]. SNPs in the XRCC3 gene have been correlated with increased risk of glioma, [22,23] as have SNPs in BRCA1[24] and EME1 [25]. Identification of these risk SNPs and the mechanisms by which they increase glioma risk may provide novel targets for new therapies in the future.

### Therapeutic significance: induced HR deficiency mediates tumor sensitization to poly (ADP-ribose) polymerase 1 (PARP1) inhibition

**Figure 1:**
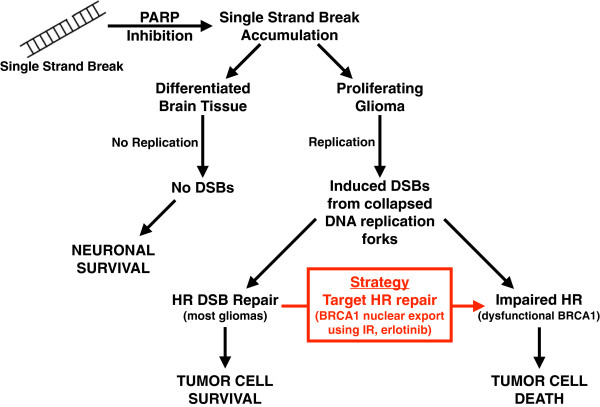
**Targeting HR repair with poly (ADP-ribose) polymerase inhibition results in tumor-specific synthetic lethality. **Model depicting potential targeting of HR repair to increase the therapeutic index of anti-glioma treatment. Sequestration of BRCA1 to the cytoplasm inhibits repair of DSBs and sensitizes cells to DNA-damaging agents. Following DNA damage, BRCA1 facilitates the repair of DNA in the nucleus. By targeting BRCA1 subcellular location, tumor cells retain unrepaired damaged DNA and are subsequently sensitized to poly (ADP-ribose) polymerase inhibitors.

Given the replication demands in proliferating tumor cells, the HR pathway, which functions during S/G2, may be a valuable target for new and high-therapeutic index GBM treatment regimens. DNA single-strand breaks (SSBs) can lead to DSBs at the replication fork. Unrepaired DSBs are lethal to proliferating cells. Dysfunction in the repair of both SSBs and DSBs would be synthetically lethal. The enzyme poly(ADP-ribose) polymerase 1 (PARP1) plays a key role in the repair of SSBs,[26] while the tumor suppressor BRCA1 is essential for HR-mediated repair of DSBs [27,28]. PARP1 inhibitors, including Olaparib, target cancers which are deficient in the repair of DSBs, exhibit up to 1,000-fold selectivity in killing BRCA1-mutated (DSB-repair deficient) cells, and provide an overall survival and progression-free survival benefit with minimal toxicity in patients with BRCA1-deficient familial breast cancer [29-33]. Unfortunately, the majorities of patients who develop sporadic tumors including malignant gliomas carry wild-type (wt) BRCA1 and are proficient in DSB repair, precluding them from this potent avenue of therapy [34]. We have previously shown that transiently exporting wt-BRCA1 protein from the nucleus (where DSBs are repaired) to the cytosol (where apoptosis is activated) makes cancer cells defective in the repair of DSBs [35]. We propose to develop an innovative therapeutic strategy that uses this export of BRCA1 from nucleus to cytoplasm to transiently convert BRCA1-proficient GBM cells into functionally BRCA1-deficient cells and thereby render them susceptible to PARP inhibitor–induced cell killing (Figure 1).

Experiments conducted in our laboratory have demonstrated proof of this concept in GBM cells. The HR pathway was inhibited by causing loss of BRCA1 function in three ways: (1) siRNA-mediated BRCA1 knockdown, (2) IR-mediated export of BRCA1 to the cytosol, and (3) erlotinib-induced BRCA1 nuclear depletion. siRNA-mediated BRCA1 knockdown results in suppression of HR repair in several cancer and non-cancer cell model systems. BRCA1 is also a nuclear-cytosolic shuttling protein, and its function is regulated by its subcellular location. When in the nucleus, BRCA1 participates in HR repair of DSBs. When in the cytosol, it enhances apoptosis. We have recently found that IR induces nuclear export of BRCA1 in response to DNA damage via the CRM1/exportin-dependent pathway. Additionally, inhibition of EGFR by erlotinib results in DNA damage and BRCA1 nuclear export [36]. In all three instances, HR-mediated DSB repair was attenuated, and tumor cytotoxicity response to the PARP inhibitor ABT-888 was significantly increased versus non-HR-inhibited controls. By inducing tumor-specific HR deficiencies, we believe that PARP inhibitor therapy will result in more efficacious, safer treatment of both HR-deficient and HR-competent tumors.

## Non-homologous End-joining (NHEJ)

While HR provides some genomic protection, NHEJ is another major DSB repair pathway in mammalian cells. In contrast to HR, NHEJ repairs on a wide variety of DSBs with distinct break structures and sequences [37] and functions predominantly during G1 when the HR repair is not available [38]. However, it demonstrates decreased fidelity compared to HR, as NHEJ repairs DSBs using no or little homologous template to ensure that the repaired strand reflects the original sequence [39].

Similarly to the HR process, NHEJ repair follows a basic motif of damaged base digestion, re-polymerization/repair, and ligation. Its details have been well-characterized by others [39]. Briefly, following identification of the DSB by the cell, Ku70 and Ku80 bind to the exposed breakpoints as a heterodimer and serve to recruit other necessary proteins [40]. DNA-dependent protein kinase (DNA-PK) is recruited to the site and exposes the DNA ends to recruited nucleases [41,42]. DNA-PK also activates the G1 DNA damage checkpoint and arrests the G1-S transition [43]. A wide variety of nucleases digest nucleotides from the DNA on both strands, and strand re-extension is facilitated by X family DNA polymerases, [44] although in a less extensive fashion than occurs in HR [11]. Finally, the DNA ligase IV complex joins the two repaired DNA ends [45].

Due to NHEJ’s role as a DNA repair system and checkpoint activator, it is no surprise that there appears to be a connection between its malfunction and tumorigenesis in the brain. SNP variations in several NHEJ genes, including those that code for the DNA ligase IV complex, [46] Ku80 and Ku70, [47] and DNA-PK’s catalytic subunit [24] have been correlated with increased risk of glioma. Furthermore, loss of DNA-PK function has been demonstrated to increase IR resistance in GBM cells [48]. Despite these implications, it may be possible to exploit the tumor’s NHEJ deficiency in planning treatment strategies.

### Therapeutic significance: NHEJ potentiation mediates IR neuroprotection

While proliferating tumor cells can repair DSBs through both HR during S/G2 and NHEJ during G1, differentiated normal neuronal cells largely rely on NHEJ to survive from DSBs [37,49]; Thus, exploration of the difference in regulation of NHEJ activities in GBM versus normal neurons may provide neuronal protection from IR-induced cytotoxicity without affording the same benefit to tumor cells, thereby improving therapeutic gain. One promising target is glycogen synthase kinase 3β (GSK3β), which regulates glucose metabolism by phosphorylating glycogen synthase and inhibits glycogen synthesis. Interestingly, we and others have recently demonstrated that GSK3β is also involved in suppression of NHEJ activity [50]. Furthermore, inhibition of GSK3β either genetically or by its specific inhibitors (lithium or SB216763) accelerates NHEJ-mediated DSB repair and protects hippocampal neurons from IR-induced apoptosis via restoration of DNA-PK-dependent NHEJ repair of DNA DSBs, [51] and attenuates neuro-cognitive toxicity in mice. Most importantly, this GSK3β-mediated radioprotection does not occur in glioma cells examined in the studies [51-53].

**Figure 2:**
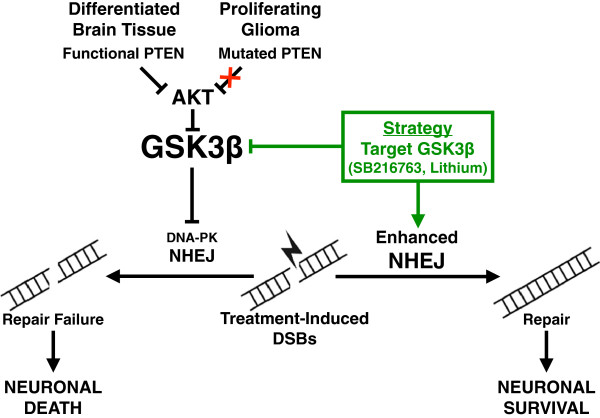
**Targeting GSK3β results in neuroprotection from IR-induced neurotoxicity. **Model depicting potential targeting of GSK3β-NHEJ signaling pathway to decrease neurotoxicity and increase the therapeutic index of anti-glioma treatment. Inhibition of GSK3β results in the upregulation of DNA-PK dependent NHEJ repair in neural but not tumor cells. By targeting GSK3β, neurons, but not GBM cells, gain enhanced DNA repair functionality, and therefore protected from IR-induced neuronal cell death.

The differential effect on NHEJ and radiation protection in normal neuron versus tumor cells may be attributed to following: (1) GSK3β is constitutively expressed at high level in differentiated cells but not expressed in proliferating cells, [54] (2) additional inhibition of GSK3β activity in proliferating tumor cells. 40% of primary gliomas demonstrate loss of phosphatase and tensin homolog (PTEN) function [55]. Canonically, PTEN inhibits the action of AKT, which in turn inhibits GSK3β [56]. As such, loss of PTEN function and /or increased AKT activity in tumor cells results in strong suppression in GSK3β activity. While most gliomas demonstrate markedly decreased GSK3β function, [57] lithium or SB216763 will not be able to generate significant modification in GSK3β-NHEJ activity as in normal neuronal cells. Together, these findings have demonstrated that the difference in GSK3β-mediated NHEJ regulation between healthy neural tissue and glioma cells can be exploited to benefit the patient, and have provided strong preclinical evidence and rational for clinical implementation of GSK3β inhibition in combination with standard GBM treatment (Figure 2).

## Future directions

Current therapies for primary brain gliomas are not effective in tumor control and often cause severe and deleterious neruo-congnitive and systemic toxicities. It is urgent to develop novel treatment with significantly improved therapeutic gains. By utilizing the strategies outlined above, it is possible to exploit differences between tumors’ and CNS cells’ DRR pathways, specifically their DSB repair mechanisms. An increased therapeutic ratio can be accomplished by either tumor-specific sensitization (BRCA1 nuclear export and HR attenuation) or by neuron-specific radioprotection (GSK3β inhibition and NHEJ potentiation). As we proceed toward genetics-driven, individualized cancer therapeutics, a deep understanding of the DRR pathways will become even more important, as they will likely harbor new treatment targets and provide new insights into how tumorigenesis occurs in the brain.

## Competing interests

The authors declare that they have no competing interests.

## Authors’ contributions

WLS & FX participated in the literature search. WLS drafted the manuscript. FX edited the manuscript. All authors read and approved the final manuscript.
